# Comparative Characterization of Ancient Wheat Cultivars Through Fatty Acid and Phytosterol Profiling

**DOI:** 10.3390/foods15122151

**Published:** 2026-06-14

**Authors:** Giuseppina Crescente, Michela Famiglietti, Francesco Siano, Giovanni Cascone, Gabriella Fasulo, Carmela Spagnuolo, Maria Grazia Volpe, Gian Luigi Russo, Stefania Moccia

**Affiliations:** 1 National Research Council, Institute of Food Sciences, 83100 Avellino, Italy; giuseppina.crescente@isa.cnr.it (G.C.); michela.famiglietti@isa.cnr.it (M.F.); francesco.siano@isa.cnr.it (F.S.); g.fasulo1984@gmail.com (G.F.); carmela.spagnuolo@isa.cnr.it (C.S.); mgvolpe@isa.cnr.it (M.G.V.); gianluigi.russo@cnr.it (G.L.R.); 2 National Agency for New Technologies, Energy and Sustainable Economic Development, 80055 Portici, Italy; giovanni.cascone@enea.it

**Keywords:** ancient wheat, lipid fraction, fatty acid profile, phytosterols, antiradical activity, GC-FID, ATR-FTIR

## Abstract

Cereal lipids influence both the nutritional value and technological properties of flours; however, their composition remains poorly characterised, particularly in ancient wheat cultivars. This study investigated the lipid fraction of flours from three ancient wheat cultivars: *Risciola* and *Carosella* (soft wheat) and *Saragolla* (durum wheat). Fatty acid and phytosterol profiles were analysed by GC-FID, while ATR-FTIR spectroscopy provided complementary spectral information. Antiradical activity was assessed by DPPH and ABTS assays. In all samples, polyunsaturated fatty acids (PUFAs) predominated (60.23–64.04% of total identified fatty acids), with linoleic acid as the major component. *Risciola* showed the highest PUFA percentage and the most favourable PUFA/SFA ratio (SFA, saturated fatty acids). β-Sitosterol was the predominant phytosterol in all cultivars, while *Saragolla* showed a higher percentage of phytostanols (campestanol and sitostanol). Exploratory multivariate analysis provided a visual overview of compositional patterns among cultivars, consistent with differences in lipid profiles within the analysed sample set. ATR–FTIR analysis supported the chromatographic findings, while antiradical assays indicated differences in radical-scavenging capacity. Overall, the combined chromatographic, spectroscopic, and antiradical approach highlights the lipid fraction as an informative descriptor of nutritional quality, cultivar-related compositional diversity, and potential functional relevance, supporting the targeted use of ancient wheat flours in cereal-based applications.

## 1. Introduction

Cereals have long played a fundamental role in human diets, providing essential nutrients and energy; accordingly, they are consistently placed at the base of dietary guidelines and food pyramids [1,2]. Within this group, wheat is one of the most important cereal crops, and wheat-based products are widely consumed worldwide [3]. Globally, the bulk of wheat cultivation, estimated at around 95%, is represented by *Triticum aestivum* L., a hexaploid species traditionally designated as “common”, “bread”, or “soft” wheat. The remainder is largely made up of *Triticum turgidum* subsp. *durum*, a tetraploid species primarily used to produce semolina for pasta production [4].

Within a balanced diet, wheat provides not only carbohydrates and proteins but also dietary fibre and a range of bioactive compounds, particularly when consumed as whole grain [5]. In cereal kernels, lipids are mainly located in the germ and outer layers, which are often removed during conventional milling, resulting in a reduced lipid content in refined flours [6]. By contrast, whole-grain flours, which preserve both the germ and the outer layers, retain a higher lipid fraction, thereby enhancing their nutritional value and contributing to their technological properties [7].

In recent years, increasing attention has been directed towards ancient wheat cultivars because of their potential role in sustainable agriculture and their distinctive nutritional profiles. These traditional cultivars are often associated with more favourable lipid profiles, including higher proportions of polyunsaturated fatty acids (PUFAs), such as linoleic and α-linolenic acids, which are linked to beneficial effects on cardiovascular health and inflammatory regulation [8,9,10]. The nutritional relevance of ω-6 and ω-3 PUFAs has been assessed using two lipid quality indices: the atherogenicity index (IA) and the thrombogenicity index (IT). In 1991, Ulbricht and Southgate highlighted that the relationship between diet and coronary heart disease is influenced not only by elevated levels of “bad cholesterol”, but also by other factors such as ω-3 and ω-6 PUFAs and monounsaturated fatty acids (MUFA) [11]. To account for these effects, they proposed IA and IT as indicators of lipid quality, reflecting the potential impact of dietary lipids on atherogenic risk and platelet aggregation. Both indices are derived from the inverse of the PUFA/SFA ratio (SFA, saturated fatty acids); therefore, higher IA and IT values indicate foods, food components, or diets with greater atherogenic and thrombogenic potential. Conversely, lower values suggest cardioprotective activity. IA and IT have been widely applied to assess the nutritional properties of foods [12,13] and are therefore adopted as complementary nutritional parameters in the present study.

Alongside fatty acids, phytosterols represent another biologically relevant component of cereal lipids. Although present in small amounts, they are widely recognised for their cholesterol-lowering effects and potential contribution to the prevention of cardiovascular and other chronic diseases [14,15]. In wheat, phytosterol levels are influenced by both botanical classification (durum vs. soft wheat) and growing conditions; however, differences in phytosterol content have also been reported between traditional and modern cultivars [16].

Despite growing interest in ancient wheat cultivars, research on their lipid fraction remains fragmentary and is mainly focused on modern cultivars or limited classes of compounds [9,17,18,19,20]. Additionally, differences in cultivar sets, geographic origin, and growing conditions often limit direct comparisons between studies [9,16]. In particular, detailed and integrated information on fatty acid composition, phytosterol and phytostanol profiles, and their compositional and nutritional relevance is largely unavailable for many traditional Italian wheats. This gap is especially evident for the soft wheat cultivars *Risciola* and *Carosella*, whose lipid composition has not previously been reported. Although these Southern Italian ancient wheats have been investigated for general physicochemical and technological traits, detailed characterisation of their lipid fraction, including fatty acids and phytosterols/phytostanols in an integrated framework, is still lacking [2]. This lack of information is particularly relevant given the increasing interest in the valorisation of local agrobiodiversity and low-input cropping systems.

In this context, the present study aimed to address this knowledge gap by providing an integrated and comparative characterisation of the lipid fraction of flours obtained from three ancient wheat cultivars (*Risciola*, *Carosella*, and *Saragolla*) grown under the same low-input agronomic conditions in southern Italy. By combining chromatographic (GC-FID) and spectroscopic (ATR-FTIR) approaches with antiradical assays, this work extends conventional compositional analysis and highlights cultivar-related differences in the lipid fraction, particularly in fatty acid and phytosterol profiles. The observed differences highlight the potential of lipid constituents not only as indicators of nutritional quality, but also as descriptors of cultivar-related compositional diversity and potential functional relevance, contributing to the valorisation of ancient wheats within sustainable food systems.

## 2. Materials and Methods

### 2.1. Raw Materials

Samples of three ancient wheat cultivars (*Risciola*, *Carosella*, and *Saragolla*) were provided by Lucifero farm in Zungoli (Avellino, Italy). *Risciola* and *Carosella* are classified as soft wheat cultivars, while *Saragolla* is a durum wheat cultivar. The grains were produced in a field trial conducted during the 2021/2022 growing season as part of a research project aimed at evaluating the agronomic sustainability and valorisation of ancient wheats in marginal areas of southern Italy under low-input cropping systems. The cultivation system included crop rotations designed to improve soil fertility and reduce dependence on intensive cereal monocultures. The grains were grown under organic farming conditions and, after harvest, the whole grains were stone-milled into wholemeal flour at Mulino Bencivenga farm in Alvignano (Caserta, Italy) under temperature-controlled conditions to minimise thermal stress during milling (https://agricoltura.regione.campania.it/psr_2014_2020/1611_2/GRADITI.html; accessed on 8 March 2026). For each cultivar, flour samples obtained from three independent field plots were analysed.

### 2.2. Particle Size Analysis

Particle size distribution was determined by laser diffraction using a Mastersizer MSS particle size analyser (Malvern Instruments Ltd., Malvern, UK). Flour samples were suspended in isopropanol, and approximately 5 mg of each sample was introduced into the small-volume cell to achieve an obscuration between 8% and 10%. Particle size distributions were calculated using the Fraunhofer model. Measurements were performed in triplicate for each sample, and the results were expressed as D50 and D90 values.

### 2.3. Lipid Extraction

Lipid extraction was performed on the three flour samples using a Soxhlet apparatus, according to AOAC International method 920.39C [21]. Specifically, 40 g of each sample was weighed into a cellulose extraction thimble and placed in the Soxhlet chamber. Diethyl ether was used as the extraction solvent. Extraction was carried out for 6 h under continuous reflux. Upon completion, the solvent was removed using a rotary evaporator (Heidolph Hei-VAP, Schwabach, Germany). Lipid content was determined gravimetrically and expressed on a dry-weight basis (g/100 g DW). Moisture content was determined as previously reported [2].

### 2.4. Antiradical Assessment of Lipid Extracts

The antiradical activity of the lipid extracts was evaluated using the DPPH and ABTS methods. Due to the lipophilic nature of the samples, the extracts were dissolved in isopropanol for both assays to achieve the following final concentrations: 0.019, 0.078, 0.3, 1.25, and 5 mg/mL. The lipid extracts were added to a DPPH solution (0.1 mM in isopropanol) and, after incubation for 20 min, the absorbance was measured at 517 nm using a Synergy HT microplate reader (BioTek, Milan, Italy).

The ABTS^•+^ solution was prepared as previously described [22]. After dilution in EtOH to reach an absorbance of 0.70 at 734 nm, the mixture was incubated for 6 min before measuring the absorbance at 734 nm (Synergy HT, BioTek). Trolox^®^ was used as a positive control (2, 4, 8, 16, and 32 µM). For each biological replicate, measurements were performed in technical triplicate. ID_50_ values were calculated based on the percentage reduction in initial absorbance observed at the various concentrations tested.

### 2.5. Preparation of Fatty Acid Methyl Esters (FAMEs)

FAMEs were prepared according to the method described by [23]. Aliquots (0.2 g) of the flour lipid extract were transferred to screw-cap Pyrex test tubes, and 2 mL of 1.25 N HCl in methanol were added. Samples were incubated in a water bath at 90 °C for 60 min. FAMEs were extracted with *n*-hexane after the addition of 2 mL of distilled water. The organic phase was filtered through 0.45 µm PVDF disposable syringe filters (EMD Millipore Corp., Billerica, MA, USA), and 1 µL was injected directly into the gas chromatograph for analysis.

### 2.6. GC-FID Analysis of FAMEs

FAMEs were analysed using a TRACE GC gas chromatograph (Thermo Scientific, Inc., San Jose, CA, USA) equipped with a Flame Ionization Detector (FID) and an SP-2560 capillary column (100 m × 0.25 mm × 0.20 μm). Samples were introduced via a split/splitless injection system of an AS 3000 autosampler, in split mode (split ratio 1:100) at 260 °C. The oven temperature program started at 140 °C (held for 5 min) and increased linearly to 260 °C (4 °C min^−1^) until the end of the analysis. The FID was maintained at 260 °C. Fatty acids were identified by comparison of retention times with a 37-component FAME standard mixture (Merck/Sigma-Aldrich, Milan, Italy), and results were reported as relative peak areas (%) of total identified FAMEs.

### 2.7. GC-FID Analysis of Sterols

The unsaponified components were extracted from the flour lipid extract as follows. 25 mL of a 2 N KOH ethanolic solution were added to 2.5 g of flour lipid extract from each sample, and the mixture was heated under reflux with magnetic stirring. Complete saponification took about 20 min after the solution clarified. Water (25 mL) was then added, and the solution was transferred quantitatively to a separatory funnel, where three extraction steps were performed with diethyl ether (40 mL). The ether phases were pooled and washed several times with water (50 mL each) until neutralized and finally dried over sodium sulphate. After filtration, the solvent was evaporated under vacuum at 30 °C. The dried samples were resuspended in *n*-hexane (2.5 mL) and analysed using the above-mentioned GC-FID equipped with an RTX-5 column (30 m × 0.25 mm × 0.25 μm; Restek, Bellefonte, PA, USA). Samples (1 μL) were introduced through the autosampler in 1:10 split mode at 250 °C. The oven temperature program started at 200 °C (held for 2 min) and increased linearly to 300 °C (20 °C min^−1^) until the end of the analysis. A plant sterol mix (Matreya, State College, PA, USA) was used as an external standard for qualitative and quantitative determinations. Data were recorded and processed using ChromQuest 5.0 software (Thermo).

### 2.8. ATR-FTIR Analysis

The Attenuated Total Reflection-Fourier Transform Infrared (ATR-FTIR) spectra of the lipid extracts from the flour samples and the unsaponified components were recorded at a resolution of 8 cm^−1^ with 32 scans in the mid-IR region (4000–650 cm^−1^), using a Spectrum 400 spectrophotometer (PerkinElmer, Waltham, MA, USA). Briefly, 7 µL of each sample was placed on the surface of the germanium crystal and allowed to dry at room temperature. The spectra were recorded, and the crystal surface was cleaned after each spectral collection with 0.1% (*w/v*) Alconox solution (Alconox Inc., New York, NY, USA). Spectral acquisition was performed in technical triplicate for each biological replicate to assess repeatability, and the average spectra were used. The background spectrum was measured against air. Spectra were processed using PE Spectrum software version 10.5.1, supplied with the instrument.

### 2.9. Indices of Lipid Quality

Nutritional quality, based on fatty acid composition, was determined by applying the following formulas reported by [11]:

Index of atherogenicity (IA): IA = [aS′ + bS″ + cS‴]/[dP + eM + fM′], with S′ = C12:0 (lauric acid); S″ = C14:0 (myristic acid); S‴ = C16:0 (palmitic acid); P = sum of n-3 and n-6 PUFA; M = C18:1 (oleic acid); M′ = sum of all remaining MUFA; a-f are empirical constants with b = 4 and all the others equal to 1.

Index of thrombogenicity (IT): IT = mSiv/[nM + oM′ + p(n-6) + q(n-3) + n-3/n-6], with Siv = sum C14:0 (myristic acid) + C16:0 (palmitic acid) + C18:0 (stearic acid); n-6 = n-6 PUFA; n-3 = n-3 PUFA; M = C18:1 (oleic acid); Mʹ = sum of all remaining MUFA; m-q are empiric constant where m = 1; n, o and *p* = 0.5; and q = 3.

### 2.10. Statistical and Multivariate Analysis

Data are presented as mean ± standard deviation (SD) of three biological replicates (*n* = 3), corresponding to flour samples obtained from independent plots. Statistical analyses were performed using SigmaPlot 15.0 software. Differences between groups were assessed using one-way ANOVA followed by Tukey’s post hoc test. When the data did not meet the assumptions required for parametric tests, the Kruskal–Wallis test followed by Dunn’s post hoc test was applied. Exact overall *p*-values were reported for statistical comparisons, and effect size was expressed as eta squared (η^2^) for one-way ANOVA. A *p*-value < 0.05 was considered statistically significant.

Principal Component Analysis (PCA) was performed as an exploratory multivariate approach to visualize compositional patterns among the three wheat cultivars based on their fatty acid and phytosterol profiles. Separate PCA models were generated for each class of compounds. Before PCA, variables were autoscaled by mean-centring and scaling to unit variance to make compounds present at different abundance levels comparable. The analyses were carried out in R version 4.6.0 RC using the FactoMineR package, while factoextra and ggplot2 were used for graphical visualization. PCA biplots were generated to display both sample distribution and the contribution of individual chemical variables. Ellipses were included only as a graphical aid to visualize the grouping of replicates belonging to the same cultivar.

Heatmaps with hierarchical clustering were generated using ClustVis, custom edition (https://biit.cs.ut.ee/clustvis/; accessed on 8 May 2026) to visualize the relative distribution patterns of fatty acids and phytosterols between wheat flour samples. Data were row-scaled prior to visualization, and the colour scale indicates relatively higher or lower values for each compound after scaling.

## 3. Results and Discussion

### 3.1. Particle Size Distribution and Lipid Content of Flour Samples

The particle size distribution is expressed as D50 and D90, representing the particle diameters below which 50% and 90% of the total particle volume are distributed, respectively. The soft wheat cultivars *Risciola* and *Carosella* exhibited a finer particle size distribution (D50: 150–210 µm; D90: 400–500 µm), whereas the durum wheat cultivar *Saragolla* showed a coarser particle size distribution (D50: 260–320 µm; D90: 550–650 µm).

Lipid content, expressed on a dry-weight basis (DW), ranged from 1.95 ± 0.07 to 2.55 ± 0.38 among the three ancient wheat flours analysed. *Risciola* showed the highest value of lipid content (2.55 ± 0.38 g/100 g DW), followed by *Saragolla* (2.02 ± 0.29 g/100 g DW) and *Carosella* (1.95 ± 0.07 g/100 g DW), as reported in Table 1, although the differences between cultivars were not statistically significant.

As all samples were grown in the same area and under identical agronomic conditions, the observed values provide a comparative description of the cultivars within the experimental context analysed, without allowing us to distinguish the relative contributions of genotype and environment.

### 3.2. Antiradical Activity

**Figure 1:**
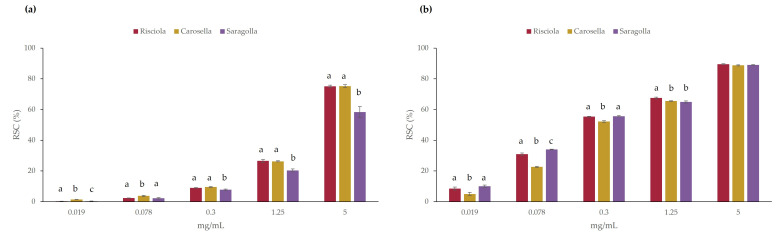
Antiradical activity of lipid extracts from three ancient wheat cultivars (*Risciola*, *Carosella*, and *Saragolla*) assessed by DPPH (**a**) and ABTS (**b**), expressed as RSC (%). Values are presented as mean ± SD. Different letters indicate statistically significant differences (*p* < 0.05). The absence of letters indicates non-significant differences between samples. Exact overall *p*-values and η^2^ for comparisons among cultivars at each concentration are provided in Table S1.

The antiradical activity of lipid extracts from three ancient wheat cultivars (*Risciola*, *Carosella*, and *Saragolla*) was evaluated using the DPPH^•^ and ABTS^•+^ assays. As shown in Figure 1a,b, both tests revealed a dose-dependent increase in Radical Scavenging Capacity (RSC). However, the response profiles and cultivar-related differences varied depending on the assay used. In the DPPH assay (Figure 1a), only weak antiradical activity was detected at concentrations below 1.25 mg/mL, with RSC values remaining below 10%. A marked increase was observed only at the highest dose tested (5 mg/mL), where *Risciola* and *Carosella* achieved over 70% inhibition, while *Saragolla* was slightly lower (58.36%). These differences were reflected in the ID_50_ values, which were substantially higher for *Saragolla* (4.18 mg/mL) than for *Risciola* (3.20 mg/mL) and *Carosella* (3.18 mg/mL), indicating lower scavenging efficiency in the DPPH system. Conversely, the ABTS assay showed uniformly high activity across all samples, with complete scavenging at the highest concentration and ID_50_ values ranging from 0.29 mg/mL (*Saragolla*) to 0.38 mg/mL (*Carosella*), indicating only minimal differences among the three cultivars (Figure 1b).

The different responses observed between the two assays can be attributed to several methodological and chemical factors, including radical chemistry, solvent-dependent reactivity, and reaction kinetics [24,25]. DPPH^•^ is mainly soluble in organic solvents, whereas ABTS^•+^ can be used in both organic and aqueous media [24,25,26]. The DPPH^•^ radical is highly stable and has significant steric hindrance around its reactive site, which may affect its reactivity towards antioxidant molecules [25]. Conversely, the ABTS assay can detect a broader spectrum of radical-scavenging compounds, including both lipophilic and hydrophilic antioxidants, and may yield higher responses depending on the sample matrix [24,26]. This methodological complementarity helps explain why ABTS produced consistently high scavenging values, while DPPH revealed differences between samples with moderate or selective radical-scavenging profiles. Overall, the combined use of both assays provides complementary evidence of the radical-scavenging activity of the lipid extracts and highlights assay-dependent differences between cultivars.

However, although numerous studies have investigated the antiradical activity of hydroalcoholic or phenolic extracts of ancient cereals, research focusing on their lipid fraction remains limited, which prevents direct comparisons with other findings [19,20].

### 3.3. Fatty Acid Profiles

Table 2 summarizes the fatty acid profiles of the lipid extracts from *Risciola*, *Carosella*, and *Saragolla* as relative peak areas (%), including the sums of SFA (ΣSFA), MUFA (ΣMUFA), and PUFA (ΣPUFA), as well as the ratio of polyunsaturated to saturated fatty acids (PUFA/SFA ratio), the ω-6/ω-3 ratio, and the IA and IT indices. Representative GC-FID chromatograms are shown in Figure S1.

All samples exhibited a typical cereal lipid profile, characterized by a predominance of PUFAs, which accounted for approximately 60.23–64.04% of the total fatty acids identified. Linoleic acid (C18:2) was the major component, followed by oleic acid (C18:1) as the main MUFA. Although the qualitative fatty acid composition was similar among cultivars, differences appeared in the relative distribution of lipid classes, indicating cultivar-related compositional differences. In particular, *Risciola* showed the highest ΣPUFA percentage (64.04 ± 0.14) and the lowest proportion of ΣSFA (16.38 ± 0.12), resulting in the most favourable PUFA/SFA ratio (3.91). In contrast, *Carosella* and *Saragolla* showed higher ΣSFA values (19.00 ± 0.89 and 18.61 ± 0.81, respectively) and slightly lower ΣPUFA percentage (60.71 ± 1.20 and 60.23 ± 1.15, respectively). This pattern suggests a slight favourable fatty acid nutritional profile for *Risciola*, and may reflect cultivar-specific metabolic traits under low-input conditions. The main drivers of these differences were the relative percentages of palmitic acid (C16:0) and the two major PUFAs, linoleic acid (C18:2) and α-linolenic acid (C18:3). In particular, *Carosella* showed the highest ω-6/ω-3 ratio (15:1), compared with *Saragolla* (12:1) and *Risciola* (13:1), mainly due to its lower α-linolenic acid content.

These findings align with a recent study on a broader panel of 22 durum wheat genotypes, including *Saragolla*, which identified linoleic acid as the predominant fatty acid and oleic acid as the main MUFA, with IA and IT values in a similar range [9]. Within the analysed set, differences in fatty acid distribution were clearly reflected in the PUFA/SFA ratio and, to a limited extent, in the IA and IT indices (Table 2). In human nutrition, a PUFA/SFA ratio of at least 0.4 is commonly recommended, with values around 1.0–1.5 considered favourable for reducing cardiovascular risk, and higher PUFA/SFA ratios are associated with greater reductions in LDL cholesterol [27,28]. Similarly, the IA and IT values ranged between 0.18–0.21 and 0.30–0.37, respectively, without significant differences among the three ancient wheat cultivars analysed. The slightly higher IT values observed in *Carosella* and *Saragolla* compared with *Risciola* are likely attributable to their lower percentage of total ω-6 fatty acids (Table 2). Overall, the data reported indicate modest differences in the lipid quality indices of the three ancient wheat cultivars investigated. Despite these limited variations, it is noteworthy from a health perspective that the IA and IT values of the ancient wheat cultivars fall within the range reported for foods recognised as important sources of ω-3 fatty acids, such as fish and other seafood [13]. Taken together, IA and IT serve as valuable indices for assessing the potential impact of fatty acid composition on cardiovascular health. Lower values of these indices indicate better nutritional quality and may contribute to reducing cardiovascular risk. However, health agencies have not yet established official reference values for IA and IT, which limits their current application in dietary guidelines. Further validation of these indices in nutritional studies establishing clear cause-effect relationships will be essential for their broader use in public health recommendations. Finally, although this study focused on compositional and nutritional descriptors of the lipid fraction, the high proportion of polyunsaturated fatty acids observed in the analysed cultivars, particularly linoleic acid, may also be relevant for lipid oxidative stability during storage and after milling [7].

### 3.4. Phytosterol Profiles

Table 3 reports the phytosterol composition of the unsaponifiable fraction of the lipid extracts as relative peak areas (%), with representative GC–FID chromatograms shown in Figure S2. Overall, β-sitosterol was the predominant sterol in all samples (38.45–48.37% of total phytosterols identified), consistent with the typical phytosterol profile reported for wheat [29]. The two soft wheat cultivars (*Risciola* and *Carosella*) showed higher β-sitosterol percentages than *Saragolla* (durum wheat), while *Saragolla* displayed a clear enrichment in phytostanols, particularly campestanol (14.42% ± 1.58) and sitostanol (15.67% ± 0.51). In addition to β-sitosterol, cultivar-dependent differences were also observed for campesterol (12.56–15.09%), whereas Δ^5^-avenasterol and stigmasterol were minor constituents and did not show significant variations between samples. This sterol distribution indicates that campestanol and sitostanol are distinctive compositional features of *Saragolla* within the analysed set. The phytostanol-rich profile may reflect cultivar-specific metabolic traits and may be of nutritional interest, given the well-documented cholesterol-lowering effects of phytostanols.

**Table 3:** Phytosterol profile of three ancient wheat cultivars (*Risciola*, *Carosella*, and *Saragolla*), expressed as relative peak area (% of total identified phytosterols).

Phytosterol (Area %)	*Risciola*	*Carosella*	*Saragolla*	Overall *p*-Value	η^2^
Campesterol	12.75 ± 0.45 ^a^	15.09 ± 0.05 ^b^	12.56 ± 0.57 ^a^	0.0005	0.9186
Campestanol	7.72 ± 0.51 ^a^	6.41 ± 0.44 ^a^	14.42 ± 1.58 ^b^	0.0001	0.9493
Stigmasterol	1.91 ± 0.16 ^a^	1.57 ± 0.19 ^a^	1.99 ± 0.20 ^a^	0.0661	0.5955
β-sitosterol	45.89 ± 1.37 ^a^	48.37 ± 0.65 ^a^	38.45 ± 2.78 ^b^	0.0014	0.8885
Sitostanol	10.78 ± 0.39 ^a^	8.84 ± 0.37 ^b^	15.67 ± 0.51 ^c^	0.000003	0.9853
Δ^5^-Avenasterol	4.56 ± 0.16 ^a^	4.17 ± 0.64 ^a^	3.68 ± 0.28 ^a^	0.1034	0.5300
Others	16.40 ± 0.76 ^a^	15.54 ± 0.30 ^a^	13.23 ± 1.30 ^b^	0.0115	0.7743

Values are reported as mean ± SD (*n* = 3, biological replicates). Different superscript letters within the same row indicate significant pairwise differences according to Tukey’s multiple comparisons test. Overall *p*-values and η^2^ refer to the one-way ANOVA.

The identified phytosterols share a common sterol nucleus but differ in their side-chain structure and degree of saturation. Sitostanol and campestanol are the saturated forms of their respective sterols, lacking the Δ^5^ double bond [30]. In plants, they share the initial steps of sterol biosynthesis up to cycloartenol, after which their pathways diverge [31]. In humans, by contrast, phytosterols are not synthesised and must be obtained from the diet; in the intestine, they compete with dietary cholesterol for absorption [32]. When consumed in relatively high doses, they have been shown to reduce serum LDL cholesterol levels [33,34]. Phytosterols also possess anticancer, anti-inflammatory, and antioxidant activities [35]. In this context, the phytostanol-rich composition observed in *Saragolla* may be relevant to the development of cereal-based foods.

### 3.5. Multivariate Analysis of Fatty Acid and Sterol Profiles by PCA and Heatmaps

PCA and heatmap-based hierarchical clustering were applied to provide an integrated overview of the fatty acid and phytosterol profiles of the three wheat cultivars. These approaches were used as exploratory tools to summarise the main compositional patterns and to visualise the relationships among samples and lipid components. Accordingly, the resulting distributions and clustering patterns are discussed here as descriptive visual trends within the analysed sample set.

**Figure 2:**
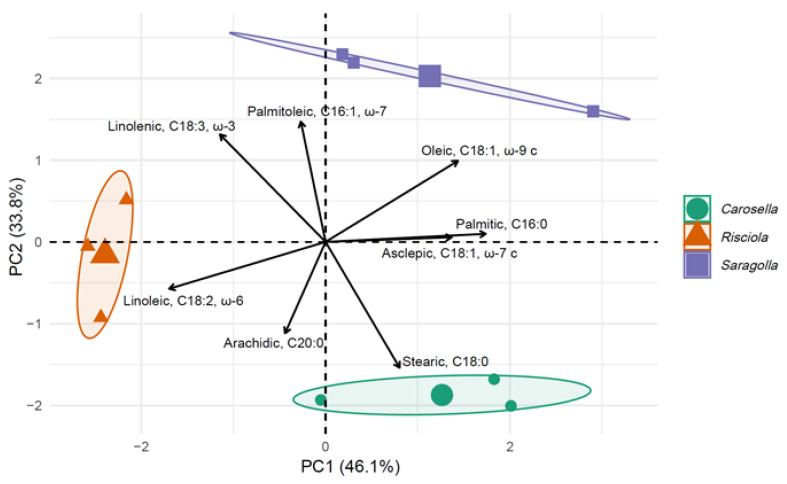
Principal Component Analysis (PCA) biplot of fatty acid composition in flours from *Risciola*, *Carosella*, and *Saragolla* wheat cultivars. Ellipses are included only as graphical aids to visualise the distribution of biological replicates belonging to the same cultivar.

For the fatty acid profile, the first two principal components explained 79.9% of the total variability, with PC1 and PC2 accounting for 46.1% and 33.8%, respectively (Figure 2). The PCA biplot provided a visual summary of the distribution of samples and variables, consistent with the univariate compositional data. *Risciola* was mainly located on the negative side of PC1, in the direction of linoleic acid (C18:2) and linolenic acid (C18:3), in agreement with its higher ΣPUFA percentage. Conversely, *Carosella* and *Saragolla* were positioned on the positive side of PC1, although they occupied different positions along PC2. *Carosella* was mainly distributed towards negative PC2 values, in the direction of stearic acid (C18:0), whereas *Saragolla* was positioned towards positive PC2 values, in the direction of oleic acid (C18:1) and palmitoleic acid (C16:1). Overall, this exploratory distribution was consistent with the compositional differences observed among the three cultivars, with *Risciola* showing a higher PUFA contribution and *Saragolla* a greater relative contribution of MUFA.

**Figure 3:**
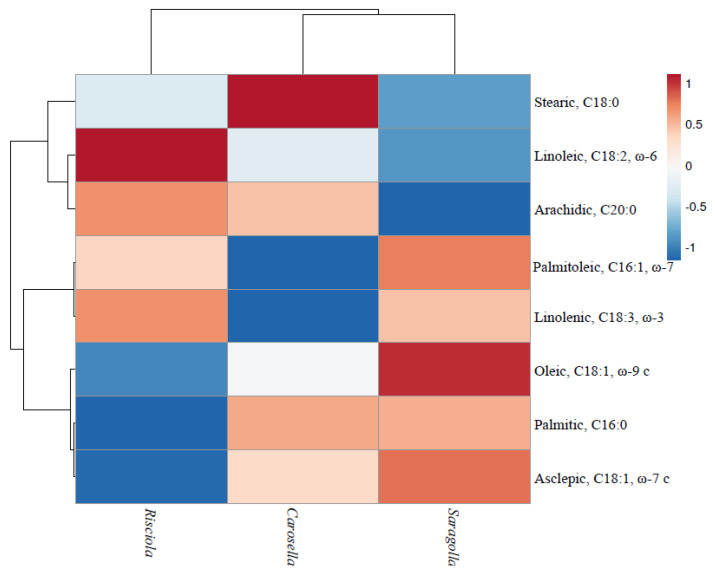
Heatmap with hierarchical clustering of fatty acid composition in flours from *Risciola*, *Carosella*, and *Saragolla* wheat cultivars.

The heatmap of fatty acid composition provided a complementary visualisation of the same compositional trends after row scaling (Figure 3). In the hierarchical clustering visualisation, the samples were grouped according to their fatty acid distribution, with *Risciola* showing a different relative pattern, mainly reflecting its higher relative percentage of linoleic acid. At the same time, the heatmap highlighted the compound-specific differences between *Carosella* and *Saragolla*, particularly the higher relative values for stearic acid in *Carosella* and for oleic and palmitoleic acids in *Saragolla*.

**Figure 4:**
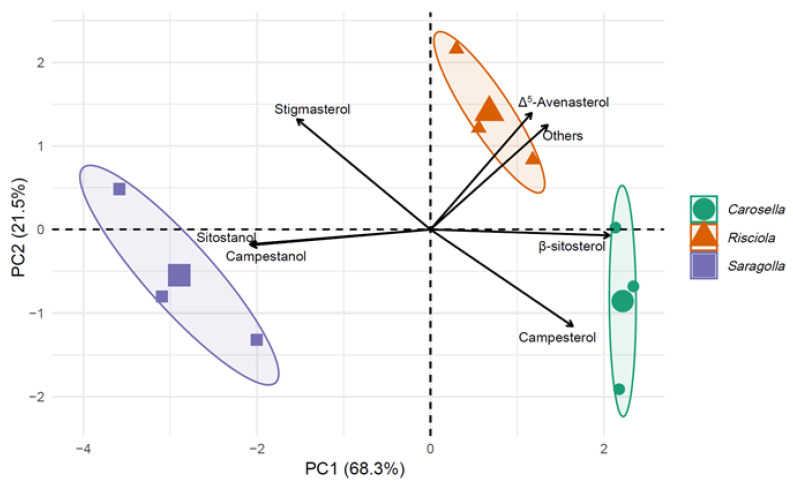
Principal Component Analysis (PCA) biplot of phytosterol composition in flours from *Risciola*, *Carosella*, and *Saragolla* wheat cultivars. Ellipses are included only as graphical aids to visualise the distribution of biological replicates belonging to the same cultivar.

For the phytosterol profile, the first two principal components explained 89.8% of the total variability, with PC1 accounting for 68.3% and PC2 for 21.5% (Figure 4). Compared with the fatty acid PCA, the phytosterol PCA provided a complementary visual summary of the sample distribution, mainly along PC1. *Saragolla* was positioned on the negative side of PC1, towards campestanol and sitostanol, consistent with the higher percentages observed for these compounds. In contrast, *Carosella* was located on the positive side of PC1, in the direction of β-sitosterol and campesterol. *Risciola* was distributed mainly along PC2, towards of Δ^5^-Avenasterol and the residual sterol fraction.

**Figure 5:**
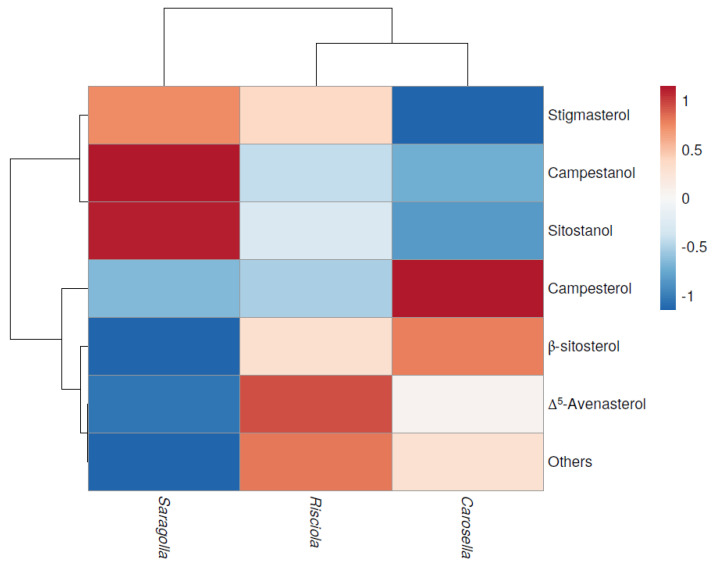
Heatmap with hierarchical clustering of phytosterol composition in flours from *Risciola*, *Carosella*, and *Saragolla* wheat cultivars.

The phytosterol heatmap provided a complementary visualisation of the relative sterol patterns observed in the PCA (Figure 5). In particular, *Saragolla* showed higher relative values for campestanol and sitostanol and lower relative values for β-sitosterol. In the hierarchical clustering visualisation, *Risciola* and *Carosella* were grouped together, although *Carosella* showed higher relative values for campesterol and β-sitosterol, whereas *Risciola* showed higher relative values for Δ^5^-Avenasterol and the “Others” fraction.

Taken together, PCA and heatmap analyses provided complementary descriptive information on the organisation of selected lipid components in the three wheat cultivars. Within this exploratory framework, fatty acid composition suggested a different relative pattern for *Risciola* compared with the other cultivars, while the phytosterol profile suggested a different relative pattern for *Saragolla* compared with *Risciola* and *Carosella*.

### 3.6. ATR-FTIR Spectroscopy

In addition to the chromatographic analyses, the lipid and phytosterol fractions were subjected to ATR-FTIR analysis. This approach enabled a relative comparison among samples based on band intensities, highlighting spectral differences consistent with compositional variability.

**Figure 6:**
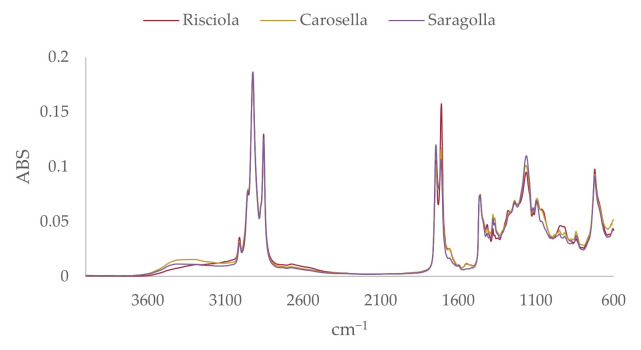
ATR-FTIR spectra of lipid fractions extracted from three ancient wheat varieties (*Risciola*, *Carosella*, and *Saragolla*).

Figure 6 shows the spectra of the lipid fractions for the three samples. The identification of functional groups was based on the ATR-FTIR bands attributed to stretching and bending vibrations. Lipid FTIR spectra display characteristic bands corresponding to the functional groups in triglycerides, fatty acids, and phospholipids. The main bands are listed in Table 4, with their position in cm^−1^, the type of vibration, and the corresponding spectral assignment.

**Table 4:** Main functional groups of lipid fractions extracted from *Risciola*, *Carosella*, and *Saragolla* samples.

Frequency (cm^−1^)	Type of Vibration	Assignment	Indications
~3006	=C–H stretching	*cis* double bond (–CH=CH–)	Presence of unsaturated fatty acids
~2920	Asymmetric C–H stretch	CH_2_ (alkyl chain)	Length of aliphatic chains
~2850	Symmetric C–H stretch	CH_3_	Terminal CH_3_ groups of aliphatic chains
~1740	C=O stretching	Esters (carbonyl group)	Triglycerides or fatty acid esters
~1715	C=O stretching	Acids (carbonyl group)	Carboxylic acid bond
~1375	CH_3_ bending	CH_3_	Presence of terminal methyl groups
~1235–1160	C–O stretching	Esters (C–O–C)	Ester bonds in triglycerides
~722	CH_2_ rocking	Ordered long chains	Crystallinity or order in the chains

The band observed around 3006 cm^−1^ indicates the presence of cis double bonds (–CH=CH–), reflecting the degree of unsaturation in the samples and supporting the presence of unsaturated fatty acids [36]. Among the three cultivars, *Risciola* showed the most intense absorption at this position, consistent with the highest PUFA percentage observed by GC-FID analysis. The intensity ratio of the bands at 2920 and 2850 cm^−1^ was also evaluated. These bands arise from the asymmetric and symmetric C–H stretching vibrations of aliphatic chains and may be influenced by factors such as chain length, conformational order, molecular packing, and the overall composition of the lipid matrix [37]. Therefore, the observed differences among cultivars (1.44 for *Risciola*, 1.49 for *Carosella*, and 1.48 for *Saragolla*) should be interpreted as indicative of subtle variations in the organisation and composition of the lipid fraction, rather than as a direct measure of branching or saturation. Nevertheless, the trend observed was consistent with the compositional differences revealed by GC-FID analysis. Regarding the composition of triglycerides, the band at ~1740 cm^−1^ is characteristic of triglycerides, while the band at ~1714 cm^−1^ is typical of carboxylic acid stretching [38,39]. All samples exhibited both bands, though in varying proportions. *Risciola* and *Carosella* displayed a more intense band associated with the carboxylic acid functional group, whereas *Saragolla* showed a stronger signal at ~1740 cm^−1^. This interpretation is further supported by the band at around 1239 cm^−1^, which is associated with ester bonds commonly found in triglycerides [40]. The band at ~722 cm^−1^, attributed to CH_2_ rocking, is typically associated with more ordered packing of long aliphatic chains and may therefore indicate a higher degree of chain organisation.

**Figure 7:**
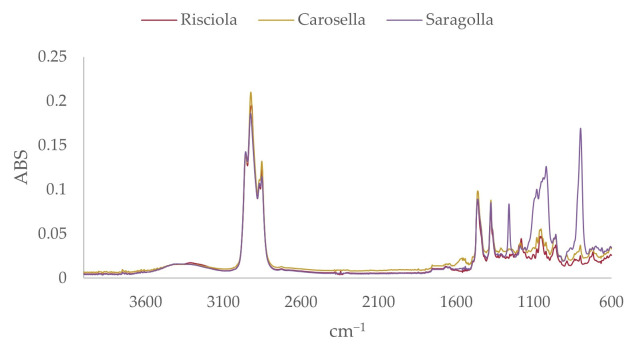
ATR-FTIR spectra of phytosterol fractions extracted from three ancient wheat cultivars (*Risciola*, *Carosella*, and *Saragolla*).

Figure 7 presents the ATR-FTIR spectra of the phytosterol fractions, and Table 5 summarises the principal bands with their wavenumbers (cm^−1^), vibrational assignments, and interpretations.

ATR-FTIR spectra of phytosterols highlighted spectral differences among the wheat cultivars analysed. A broad, weak absorption band around 3400 cm^−1^ indicated the presence of hydroxyl groups, characteristic of the alcohol functional group common to sterols. This signal appeared in all samples due to the –OH group in sterol molecules [41]. The band near 1640 cm^−1^ has been attributed to the stretching vibration of C=C double bonds. This feature was particularly associated with unsaturated sterols such as stigmasterol, which contains double bonds in the steroid nucleus or side chain. The low intensity of this band was consistent with the low percentage of stigmasterol observed by GC-FID. The absorption bands around 2930 cm^−1^ and 2860 cm^−1^, together with those at 1465 cm^−1^ and 1375 cm^−1^, arise from C–H stretching and bending vibrations within the hydrocarbon moieties of sterol molecules. These regions indicate the aliphatic structure of the steroid nucleus and its side chains. The *Carosella* sample exhibited the strongest signals in these regions, suggesting a greater contribution of aliphatic moieties in this fraction. Notably, the region between 870 and 800 cm^−1^ showed absorption associated with out-of-plane C–H bending vibrations, related to the unsaturated tetracyclic ring system characteristic of some phytosterols. This region was particularly pronounced in the *Saragolla* sample and may therefore reflect its distinctive sterol profile. Using ATR-FTIR spectroscopy, it is not possible to distinguish individual fatty acids and sterols in the analysed samples. However, the technique can provide complementary spectral information consistent with differences in fatty acid and phytosterol composition. Overall, the spectroscopic differences observed among cultivars, consistent with the chromatographic results, suggest that the qualitative profile of the lipid fraction may contribute to the compositional and nutritional characterisation of ancient wheat flours.

This work combines compositional GC-FID profiling with ATR-FTIR as a rapid complementary tool, addressing the frequent underappreciation of endogenous wheat lipids despite their functional relevance [42].

## 4. Conclusions

This study provides an integrated compositional and functional assessment of the lipid fraction in flours from selected ancient wheat cultivars, *Risciola*, *Carosella*, and *Saragolla*, grown under uniform agronomic conditions. Although total lipid content was similar among samples, cultivar-related differences were observed in fatty acid composition and phytosterol profiles, highlighting the importance of qualitative rather than quantitative lipid traits. These findings are consistent with previous research indicating that lipid and bioactive compound profiles in wheat may be influenced by genotype and growing conditions [43].

*Risciola* exhibited a more favorable fatty acid profile, with the highest PUFA percentage and the most favorable PUFA/SFA ratio, while *Saragolla* was distinguished by a higher percentage of phytostanols, particularly campestanol and sitostanol, suggesting a distinctive sterol pattern within the analyzed sample set. These cultivar-dependent differences indicate informative lipid traits with potential as nutritional indicators and as descriptors of cultivar-related compositional diversity, in line with [44]. Beyond compositional reporting, the study translates fatty acid profiles into IA and IT indices, enabling a standardized nutritional interpretation of cultivar-level lipid differences.

ATR-FTIR analysis provided spectral information consistent with the chromatographic data, supporting its potential use as a rapid, complementary, and non-destructive tool for describing lipid-related structural features in cereal matrices. Antiradical assays revealed measurable radical-scavenging capacity in the lipid extracts, although their discriminative power was assay-dependent, with ABTS showing uniformly high activity and DPPH highlighting differences only at higher concentrations.

Notably, this work helps address the limited information on the lipid and phytosterol profiles of these ancient wheat cultivars, providing compositional data useful for characterizing underexplored cereal biodiversity. Further targeted studies, using specific oxidation markers, would complement the current lipid characterization by specifically addressing lipid oxidative stability and clarifying how cultivar-specific fatty acid profiles may be relate to oxidation susceptibility. Overall, these findings may support the selection, valorization, and targeted use of ancient wheat flours for cereal-based food development within sustainable agri-food systems.

## Figures and Tables

**Table 1:** Lipid content (g/100 g DW) of flours from three ancient wheat cultivars (*Risciola*, *Carosella*, and *Saragolla*).

Sample	Lipid (g/100 g DW)
*Risciola*	2.55 ± 0.38
*Carosella*	1.95 ± 0.07
*Saragolla*	2.02 ± 0.29

Data are expressed as mean ± SD (*n* = 3, biological replicates). The absence of letters indicates non-significant differences between samples according to one-way ANOVA (exact *p*-value = 0.2133; η^2^ = 0.6430).

**Table 2:** Fatty acid profile of lipid extracts from three ancient wheat cultivars (*Risciola*, *Carosella*, and *Saragolla*), expressed as relative peak area (% of total identified FAMEs).

Fatty Acid (Area %)	*Risciola*	*Carosella*	*Saragolla*	Overall *p*-Value	η^2^
Palmitic, C16:0	14.75 ± 0.22 ^a^	17.17 ± 0.83 ^b^	17.13 ± 0.65 ^b^	0.0046	0.8343
Palmitoleic, C16:1, ω-7 *c*	0.22 ± 0.02 ^a^	0.14 ± 0.02 ^b^	0.24 ± 0.04 ^a^	0.0112	0.7763
Stearic, C18:0	1.35 ± 0.03 ^ab^	1.56 ± 0.07 ^a^	1.27 ± 0.14 ^b^	0.0206	0.7260
Oleic, C18:1, ω-9 *c*	18.45 ± 0.11 ^a^	19.13 ± 0.42 ^b^	19.94 ± 0.15 ^c^	0.0014	0.8878
Asclepic, C18:1, ω-7 *c*	0.93 ± 0.05 ^a^	1.02 ± 0.05 ^a^	1.05 ± 0.18 ^a^	0.4500	0.2337
Linoleic, C18:2, ω-6 *c*	59.36 ± 0.09 ^a^	56.84 ± 1.10 ^b^	55.64 ± 1.02 ^b^	0.0052	0.8268
Arachidic, C20:0	0.29 ± 0.08 ^a^	0.28 ± 0.01 ^a^	0.21 ± 0.06 ^a^	0.2872	0.3402
Linolenic, C18:3, ω-3 *c*	4.68 ± 0.05 ^a^	3.87 ± 0.10 ^b^	4.58 ± 0.15 ^a^	0.0002	0.9435
*cis*-11-Eicosenoic, C20:1, ω-9 *c*	n.d.	n.d.	n.d.		
ΣPUFA	64.04 ± 0.14 ^a^	60.71 ± 1.20 ^b^	60.23 ± 1.15 ^b^	0.0054	0.8241
ΣSFA	16.38 ± 0.12 ^a^	19.00 ± 0.89 ^b^	18.61 ± 0.81 ^b^	0.0075	0.8042
ΣMUFA	19.60 ± 0.07 ^ab^	20.29 ± 0.47 ^a^	21.24 ± 0.33 ^b^	0.0029	0.8573
PUFA/SFA ratio	3.91	3.19	3.24		
Total ω-6	59.36 ± 0.09 ^a^	56.84 ± 1.10 ^b^	55.64 ± 1.02 ^b^	0.0052	0.8268
Total ω-3	4.68 ± 0.05 ^a^	3.87 ± 0.10 ^b^	4.58 ± 0.15 ^a^	0.0002	0.9435
ω-6/ω-3 ratio	13:1	15:1	12:1		
IA	0.18	0.21	0.21		
IT	0.30	0.37	0.35		

n.d.= not detected. Values are reported as mean ± SD (*n* = 3, biological replicates). Different superscript letters within the same row indicate significant pairwise differences according to Tukey’s multiple comparisons test. Overall *p*-values and η^2^ refer to the one-way ANOVA.

**Table 5:** Main functional groups of phytosterol fractions extracted from *Risciola*, *Carosella*, and *Saragolla* samples.

Frequency (cm^−1^)	Type of Vibration	Assignment	Indications
~3400	O–H stretching (broad)	Alcohol group (–OH)	Typical of a hydroxyl group at the C3 position
~2930–2860	C–H stretching (asym./sym.)	CH_2_/CH_3_ in alkyl chains	Common in long aliphatic structures
~1465–1375	C–H bending	CH_3_ and CH_2_	Contribution of methyl/methylene in the nucleus and side chain
~1050–1020	C–O stretching	Secondary alcohol (C–O)	Vibration of the secondary alcohol group
~870–800	Out-of-plane C–H bending	Double bond in rings	Indicates the presence of cyclic rings and unsaturation
~1640–1625	C=C stretching	Alkenic double bond	Present in some sterols (e.g., stigmasterol)
~755–720	CH_2_ rocking	Long aliphatic chains	Signals associated with crystalline order

## Data Availability

The original contributions presented in the study are included in the article/Supplementary Materials, further inquiries can be directed to the corresponding author.

## Supplementary Materials

The following supporting information can be downloaded at: https://www.mdpi.com/article/10.3390/foods15122151/s1, Table S1. Overall *p*-values and effect sizes for the comparison of the antiradical activity of lipid extracts from *Risciola*, *Carosella*, and *Saragolla* wheat cultivars at the concentrations tested in the DPPH and ABTS assays; Figure S1. GC-FID chromatograms of fatty acid methyl esters (FAMEs) from three ancient wheat cultivars: (A) *Risciola*, (B) *Carosella*, and (C) *Saragolla*; Figure S2. GC-FID chromatograms of sterols from three ancient wheat cultivars: (A) *Risciola*, (B) *Carosella*, and (C) *Saragolla*.
