# Supplementary material for: Comparative Characterization of Ancient Wheat Cultivars Through Fatty Acid and Phytosterol Profiling
Source: Foods. 2026 Jun 14;15(12):2151. doi: 10.3390/foods15122151 (PMC13298389; doi:10.3390/foods15122151)
Supplement: Supplementary file 1 [file foods-15-02151-s001.zip › foods-4355344-supplementary.pdf]

Supplementary materials

Table S1. Overall p-values and effect sizes for the comparison of the antiradical activity of lipid extracts from *Risciola, Carosella,* and *Saragolla* wheat cultivars at the concentrations tested in the DPPH and ABTS assays.

**Assay**	**Concentration (mg/mL)**	**Overall p-value**	η^2
DPPH	0.019	0.0009	0.9906
	0.078	0.0022	0.8705
	0.300	0.0010	0.9014
	1.250	0.0001	0.9500
	5.00	0.0001	0.9527
ABTS	0.019	0.0078	0.8562
	0.078	< 0.000001	0.9919
	0.300	0.000089	0.9554
	1.250	0.0025	0.8642
	5.00	0.0601	0.6081

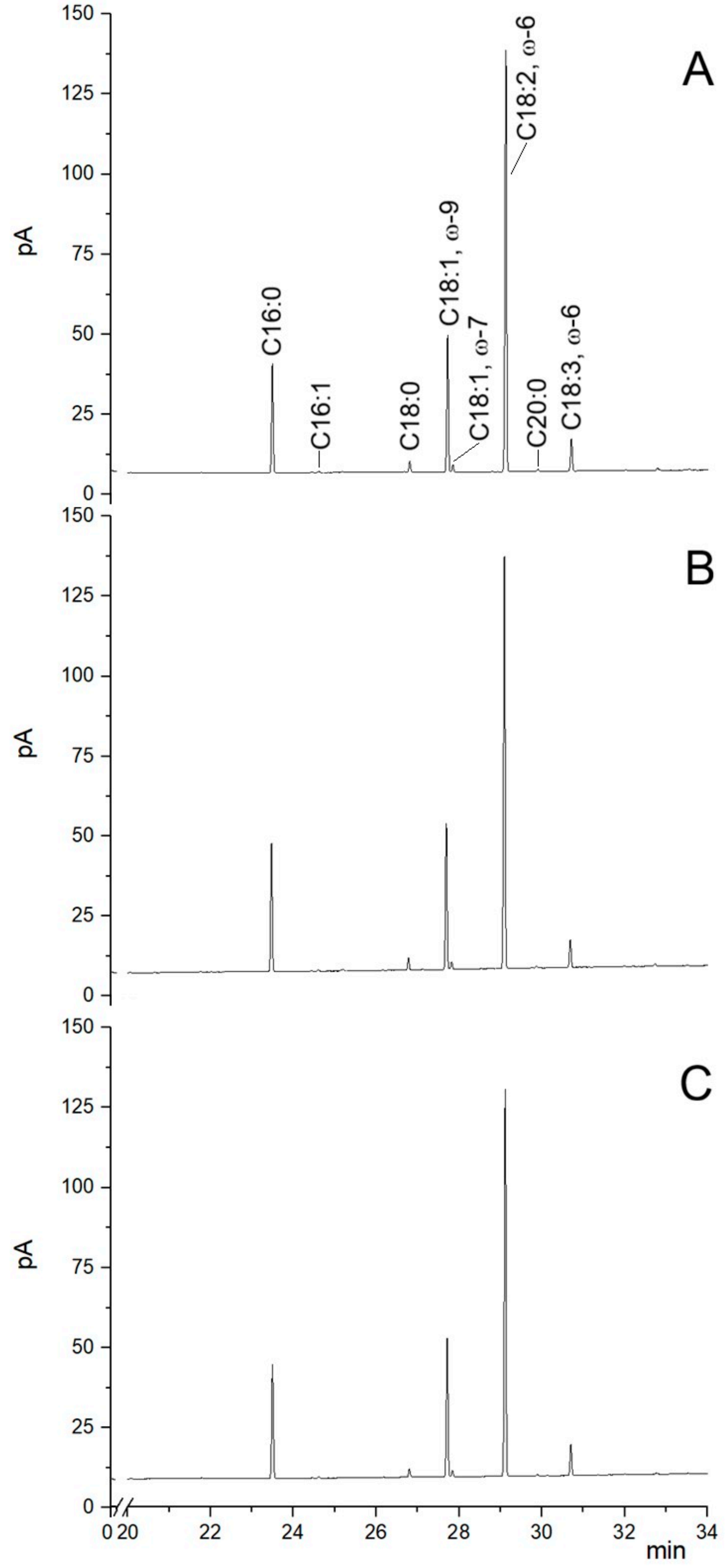

Figure S1. GC-FID chromatograms of fatty acid methyl esters (FAMEs) from three ancient wheat cultivars: (A) *Risciola*, (B) *Carosella*, and (C) *Saragolla*.

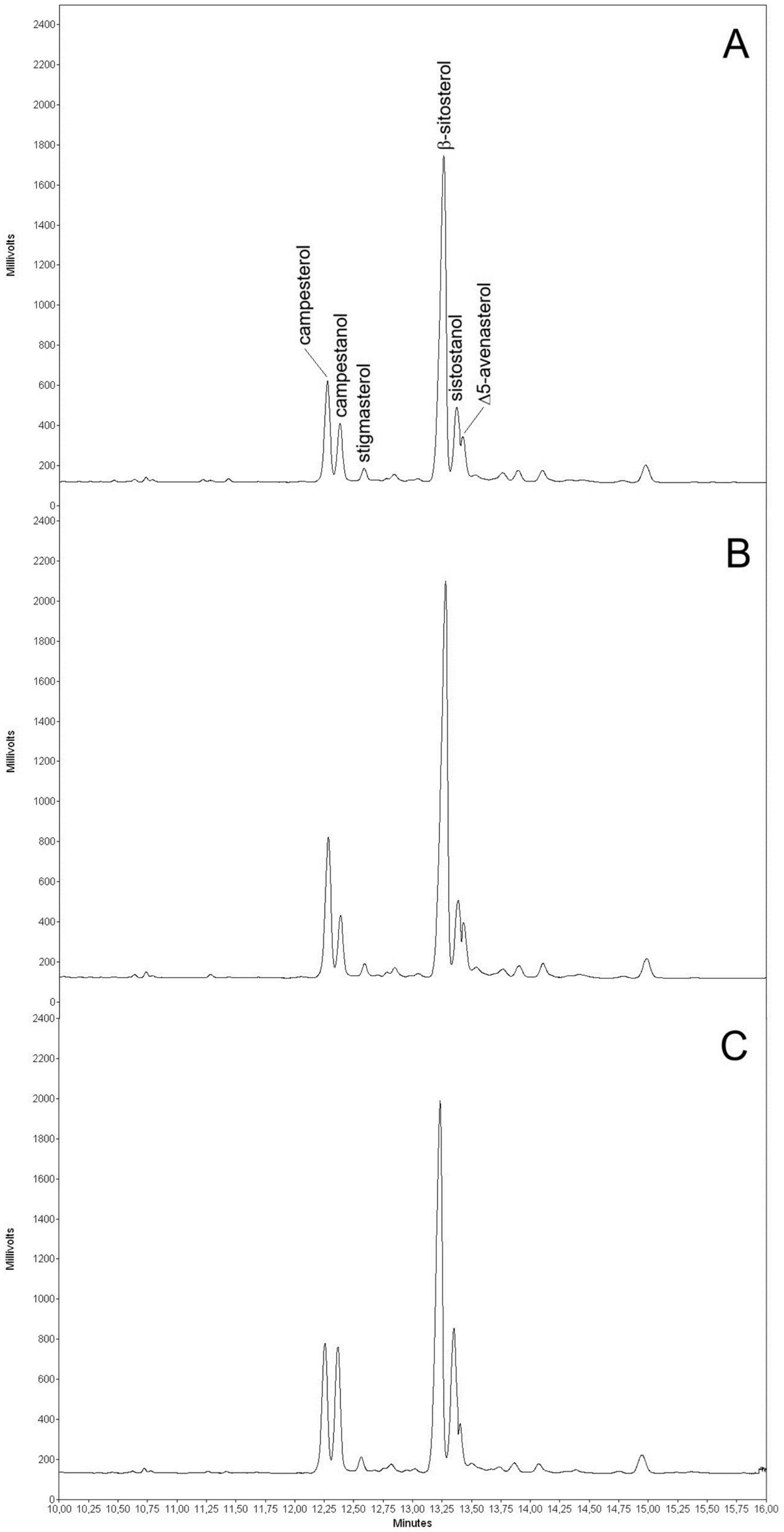

Figure S2. GC-FID chromatograms of sterols from three ancient wheat cultivars: (A) *Risciola*, (B) *Carosella*, and (C) *Saragolla*.
